# Salt tolerance of selected halophytes at the two initial growth stages for future management options

**DOI:** 10.1038/s41598-021-89462-3

**Published:** 2021-05-13

**Authors:** Fedae A. Alhaddad, Mohammed H. Abu-Dieyeh, El-Sayed Mohamed ElAzazi, Talaat A. Ahmed

**Affiliations:** 1 Department of Biological and Environmental Sciences, College of Arts and Sciences, Qatar University, P.O. Box 2713, Doha, Qatar; 2 Department of Agricultural Research, Ministry of Municipality and Environment, P.O. Box 200022, Doha, Qatar; 3 Egyptian Deserts Gene Bank, Desert Research Center, B.O.P. 11753, Cairo, Egypt; 4 Environmental Science Center, Qatar University, P.O. Box 2713, Doha, Qatar

**Keywords:** Natural variation in plants, Plant domestication, Plant ecology, Plant physiology, Environmental sciences

## Abstract

Scarcity of water and the small area of the agricultural land are considered as the crucial environmental issues challenged the Arabian Gulf countries. In this study, experiments were conducted to identify the salt tolerance during the germination and the seedling stages of some native halophytes in the State of Qatar. Seeds of eight native species (*Salsola setifera, Halopeplis perfoliata, Caroxylon imbricatum*, *Suaeda aegyptiaca*, *Acacia tortilis*, *Limonium axillare*, *Tetraena qatarensis* and *Aeluropus lagopoides*) were investigated. Except for *Tetraena qatarensis*, *Acacia tortilis* and *Suaeda aegyptiaca,* all achieved ≥ 30% of seed germination at a concentration of 200 mM NaCl. Around 30% of *Salsola setifera* seeds were able to germinate in a salt concentration of 400 mM. Germination recovery of seeds that have been treated with 800 mM NaCl for 3 weeks was the greatest for *Halopeplis perfoliata* (94%) and the lowest for *Aeluropus lagopoides* (22%). Five halophytes were investigated for seedling growth under saline irrigation ranged from 0 to 600 mM NaCl. No significant differences obtained in growth biomass of seedlings of each of *Caroxylon imbricatum, Suaeda aegyptiaca* and *Tetraena qatarensis* between saline and non-saline treatments.

## Introduction

Known for their high tolerance of salt concentrations, halophytes are plants that can tolerate what kills 99% of other species^1^. Halophytes are plants that have the ability to complete its life cycle in salt concentrations of not less than 200 mM NaCl^1,2^. Halophytes have been regarded as a rich source of potential new crops (forage, oilseed and vegetable crops) because of their diversity^3,4^. The most productive species yield around 10 to 20 ton/ha of biomass with seawater irrigation, equivalent to conventional crops^5^. The oilseed halophyte, *Salicornia bigelovii*, yields an average biomass of 18.0 ton/ha and about 2 ton/ha of seed containing 31% protein and 28% oil, similar to soybean yield and seed quality^6^. Halophytes could also be good sources of livestock feeds^7^.

In Qatar the uses of wild plants have been recoded for long period of time^8,9^. Some plants used as food for camel and other animals like *Salsola setifera*, *Caroxylon imbricatum*, *Acacia tortilis* and *Aeluropus lagopoides*. In addition, some plant species showed pharmacological properties including anti-inflammatory (*Salsola setifera and Caroxylon imbricatum*), treating tooth from gum infection (*Suaeda aegyptiaca*)^8,10^.

The demands on natural resources such as, fresh water and suitable land for agriculture have been increasing progressively worldwide. Concerns about food and water security in the arid land of Middle East countries in general and in the Gulf countries in particular have been raised^11^. For water and food security purposes, scientists were looking for innovation practices to deal with unutilized saline areas by growing salt tolerant plants that can germinate and survive in such conditions^12^. In arid environments, the scarcity of water and the sudden increase of population have increased the demand on using natural plants that can cope with the drought and saline conditions^13^. The high yield of such plants in arid environments without exhausting freshwater resources is the major objective for increasing the governmental and the scientific research efforts on the halophytes in Qatar. According to literature, few studies have been conducted about eco-physiological aspects of halophytes in Qatar^14–19^. Most of the previous work was mainly aimed to describe the saline vegetation based on saline soil habitats or plant communities^8,10,14,20^ without much consideration for their ecological and economical potential for the purpose of halophytic crop establishment.

To survive in a saline environment, halophytes need first to achieve the seed germination, which represents the most critical stage in the life cycle followed by the seedling survival stage^21,22^.The difference in response to germination among halophytes in a saline environment determine the distribution and abundance of halophyte populations^23–25^. Scientists indicated that seeds of halophytes remain viable for a prolonged period of exposure to salt stress and germinate when conditions are favorable^26,27^. Additionally, beyond the tolerance limits of the species, the high salinity can completely inhibit seed germination^28^. According to Khan and Gul (2006), the physiological response of seed germination has perhaps evolved to make the particular halophyte adapt to specific environmental conditions^29^.

The plant species, included in this study characterized to survive under harsh environmental condition of high temperature, limited annual rainfall, which in turn increases the salinity of soil and scarcity of available fresh water for irrigation. Sodium and chloride ions are the main elements that aid in increasing soil salinity^9^. The electrical conductivity (Ece) reaches up to 195 deciSiemens/meter (dS/m) in Al-khor and Al-Dhakhira areas (around 1950 mM NaCl), and it could reaches up to 71 (dS/m) in inland areas like near Qatar University (around 700 mM NaCl)^9,30^.

The present study is kind of the first in screening for local halophytes to serve future research in investigating their economical values and the utilization of the salt affected lands in coastal and in inland areas. The goal of this research is to provide quantitative data about degree of tolerance of halophytes from Qatar to saline irrigation during the two critical initial stages, seed germination and seedling survival. The results of this study may possibly contribute to a better understanding of the performance of these species, and they will allow us to recommend the best halophyte(s) for future use in vegetation restoration.

## Materials and methods

### Study site and seed collection

Qatar is located in the Arabian Gulf (25°35′48″ N and 51°18′39″ E) that characterized as warm and semi desert ecosystem. It is a peninsula occupying an area of 11,000 km^2^ and a coastline of 900 km in length with a very shallow, semi-enclosed sea characterized by hyper-salinity (39–41 psu) practical salinity unit for surface water). The main landforms of Qatar are stony desert (Hamads) and rocky ridges (together forming 87.86%), depressions (2.44%), Sabkha (6.06%) and sand dunes (around 3.12%)^10^. As a subtropical desert, Qatar is hot and has dry weather, the annual rainfall is about 81 mm. The daily average temperature ranges from 18.5 in January to 37 °C in July and the average maximum air temperature is 31 °C, although the maximum temperature could exceed 47 °C during summer months.

Seeds of eight plant species (*Salsola setifera* (Moq.) Akhani, *Halopeplis perfoliata* (Forssk.) Bunge ex Schweinf. & Asch, *Caroxylon imbricatum* (Forssk.) Moq., *Suaeda aegyptiaca* (Hasselq.) Zohary, *Acacia tortilis* (Forssk.) Hayne, *Limonium axillare* (Forssk.) Kuntze, *Tetraena qatarense* (Hadidi) Beier & Thulin and *Aeluropus lagopoides* (L.) Thwaites) were collected from different areas of the state of Qatar. All plant species are native to Qatar and not endangered or threaten species. They were collected after proper permissions and all methods were carried out in accordance with relevant guidelines and regulations. Seeds of *Acacia tortilis* and *Caroxylon imbricatum* were collected from Qatar University protected field III (25° 22′ 6″ N; 51° 29′ 35″ E) in June 2014 and October 2014 respectively. Seeds of *Aeluropus lagopoides* were collected in June 2014 from Dakhira (25° 42′ 4″ N, 51° 33′ 16″ E). *Tetraena qatarense* seeds were collected from Al-khawr (25° 37′ 18″ N, 51° 32′ 41″ E) in September 2014. *Suaeda aegyptiaca, Halopeplis perfoliata* and *Salsola setifera* seeds were collected from Al Shamal (26° 8′ 3″ N, 51° 11′ 14″ E and 26° 8′ 24″ N, 51° 13′ 13″ E) in January 2015. Seeds of *Limonium axillare* were provided by the gene bank of Department of Agricultural Research, Ministry of Municipality and Environment (collected in September 2013 from Dakhira (25°44′05″N 51°32′51″E).

Seeds were cleaned from debris, inert material and damaged and infected seeds using a magnifying lens and sieves with different mesh sizes, as recommended by Rao et al. (2006). Seeds were then air-dried, cleaned and stored in brown paper bags at room temperatures (23 ± 2 °C) for future investigation. During the storage period (1–2 months)*,* the seeds of each species were characterized for their weight, colour and dimensions and then photographed in the Scientific Photography Laboratory at Environmental Science Center of Qatar University.

### Germination experiment

The germination experiment was conducted in a growth chamber at alternating temperature regime of (23–30 °C) based on a 24-h cycle of 12-h day length, at a fluorescent light intensity of 165 ± 20 μmole m^−2^ s^−1^ and relative humidity of 70%. The above-mentioned conditions were preliminary investigated and found to be the best for the selected species. Prior to experiment, seeds were surface sterilized using 5% sodium hypochlorite solution for 20–60 s (based on seed size) and thoroughly rinsed with distilled water for 2–3 min. Based on extensive preliminary tests in the presence of distilled water, seeds of the studied species have shown variable germination potentials ranging from 60% to around 100%. Only seeds of *Acacia tortilis* have a hard seed coat, so the mechanical scarification (clipping off part of seed shell) was done for the seed to break the thick seed coat. For *Salsola setifera* naked seeds without perianths, while for *Caroxylon imbricatum* seeds with perianths were used in germination experiment (For *C. imbricatum*, preliminary investigation resulted in better germination % when the seeds have perianth compared to naked seeds). Three layers of cotton cheesecloth were placed onto the bottom of the 10 cm diameter Petri plate**.** The experiment is one factor with seven treatment levels including the control treatment (distilled water) and six different concentrations of NaCl: 50, 100, 200, 400, 600 and 800 mM NaCl. Three ml of each of the assigned treatment were transferred to the respected Petri plate. Ten seeds of each halophyte species were placed on the moistened cotton cloth in each Petri plate. The Petri plates were arranged in a completely randomized design, with five replications. Around 2–3 ml of appropriate treatment solution was applied on alternate days to each Petri plate after rinsing out the previous solution with distilled water. Seeds were considered to have germination whenever the radicle emerged. The number of germinated seeds was daily counted for around 3 weeks. The experiment was terminated whenever there was no further germination in treatments for three consecutive days. The following are the main parameters used to describe the germination of the tested plants:

(1) Final germination percentage:

$${\text{Germination}}\,\left( \% \right) = \frac{{{\text{Number}}\,{\text{of}}\,{\text{Germinated}}\,{\text{seeds}}}}{{{\text{Total}}\,{\text{number}}\,{\text{of}}\,{\text{seeds}}}} \times 100; $$

(2) The rate of germination was estimated using a modified Timson index of germination velocity $= \sum G/t$, where *G* is the percentage of seed germination at 2-day intervals and *t* is the total germination period^31^. The maximum value using this index is 50.

(3) Germination recovery. All seeds from the previous germination tests which did not germinate after 3 weeks at different salinity levels, were placed in new Petri plates with filter paper moistened with deionized water, and incubated under the same conditions for additional period to study recovery of germination. The germination recovery percentage was calculated using the following formula^28^:

$${\text{Recovery}}\,{\text{percentage}} = \frac{{\left( {a - b} \right)}}{{\left( {c - b} \right)}} \times 100$$

where ***a*** is the total number of seeds that germinated after being transferred to distilled water, ***b*** is the total number of seeds that germinated in a given saline solution and ***c*** the total number of seeds. Germination recovery was only tested for salt treatments that achieved 50% of germination and higher.

### Seedling experiments

Five halophytes species were selected (based on their high abundance and occurrence at Qatar university Campus) to be tested for seedling growth experiments: *C. imbricatum, S. aegyptiaca, A. tortilis, T. qatarensis and A. lagopoides.* This experiment was divided into two groups: Group-1 for seedlings of *C. imbricatum, S. aegyptiaca* and *A. tortilis*, they were grown from seeds, and Group-2 for seedlings of *T. qatarensis and A. lagopoides*, which were transplanted to pots from Qatar University protected field III. The seedlings were collected after proper permissions and all methods were carried out in accordance with relevant guidelines and regulations.

For Group-1, 100 seeds were sown in trays using 60% peat moss and 40% dry sandy soil. The trays were periodically irrigated with tap water and kept under greenhouse conditions. Germinated seedlings of each species (≈2 weeks post sowing) were separately transplanted in 10 cm diameter plastic pots containing natural desert soil (88% sand, 9% silt and 3% clay) and left for 4 weeks of establishment.

Sodium chloride treatments were applied to 6-week-old seedlings of: *Suaeda aegyptiaca* and *Caroxylon imbricatum* and 8-week-old seedlings of: *Acacia tortilis, Tetraena qatarensis* and *Aeluropus lagopoides.* The experiment lasted for 7 weeks for each experiment.

This experiment was laid out in a completely randomized design with 4 replications under greenhouse conditions at 35 $\pm$ 3 °C day temperature and 23 $\pm$ 2 °C night temperature, 14 h daylight and 10 h dark night. The used concentrations of salt treatments were 0, 50, 100, 200, 400 and 600 mM of NaCl. At the initial stage and prior to experimental treatments, the salt concentration was given to seedlings in an irrigation regime through gradually increasing the salt concentration as described in Table 1. The appropriate salt concentration was reached prior or at 6 days of NaCl pretreatment.

**Table 1:** The NaCl pretreatment regime of irrigation used in the halophyte seedling experiment.

First day	0 mM	0 mM	0 mM	0 mM	0 mM	0 mM
Second day	0 mM	0 mM	0 mM	0 mM	0 mM	50 mM
Third day	0 mM	0 mM	0 mM	0 mM	50 mM	100 mM
Fourth day	0 mM	0 mM	0 mM	50 mM	100 mM	200 mM
Fifth day	0 mM	0 mM	50 mM	100 mM	200 mM	400 mM
Sixth day	0 mM	50 mM	100 mM	200 mM	400 mM	600 mM

Plants were watered twice a day, one in the afternoon using the assigned NaCl treatment and the second in the early morning of the next day with tap water only to prevent salt built up in the substrate (the above-mentioned irrigation regime was chosen based on a preliminary study). The measured parameters were (1) plant height at time of treatment and 6 weeks after treatments. (2) biomass after 6-weeks of treatment. The plants were harvested and gently washed with tap water to remove soils and then cut to separate above ground from below ground parts. The washed plant parts were wrapped in tissue paper for around 30 s to remove excess of water and then the fresh weight of the above and belowground parts were measured. The above and below ground parts of each plant were placed in labeled paper bags and dried in an oven at 75 °C for 72 h. After drying, the dry weights of each part was measured and recorded. Percentage growth change in plant height was calculated based on the following equation: Percentage Change in Plant height = {(plant height at harvesting time − plant height before treatment)/plant height before treatment)} × 100%.

### Statistical analysis

All percentage data of seed germination experiments were arcsine transformed and then analyzed using One-way ANOVA to test the significant effects of the measured variables of treatments, the means were separated using Tukey’s test at* p* ≤ 0.05. Means were compared on the transformed scale and were converted back to the original scale for presentation of results. Seedling growth data were also analyzed using One-way ANOVA to test the significant effects of the measured variables of treatments, the means were separated using Tukey’s test at* p* ≤ 0.05. All statistical analyses were done using the SigmaPlot 13 (Systat Software, San Jose, CA), www.systatsoftware.com

## Results

### Seed germination experiment

**Figure 1:**
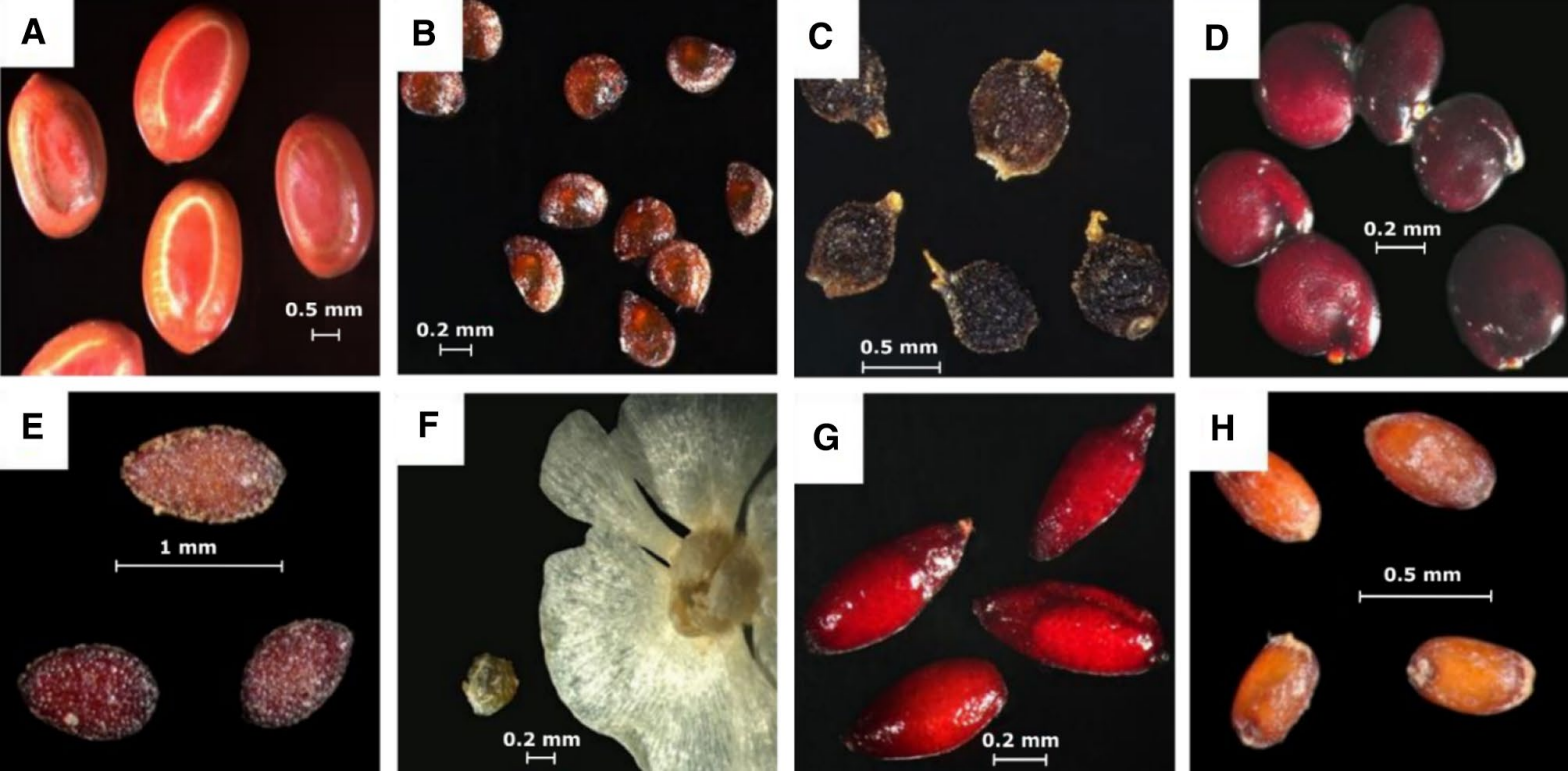
Pictures of seeds of species used in the present study: (**A**) *Acacia tortilis,* (**B**) *Halopeplis perfoliata,* (**C**) *Salsola setifera,* (**D**) *Suaeda aegyptiaca,* (**E**) *Tetraena qatarensis,* (**F**) *Caroxylon imbricatum,* (**G**) *Limonium axillare,* (**H**) *Aeluropus lagopoides.*

**Figure 2:**
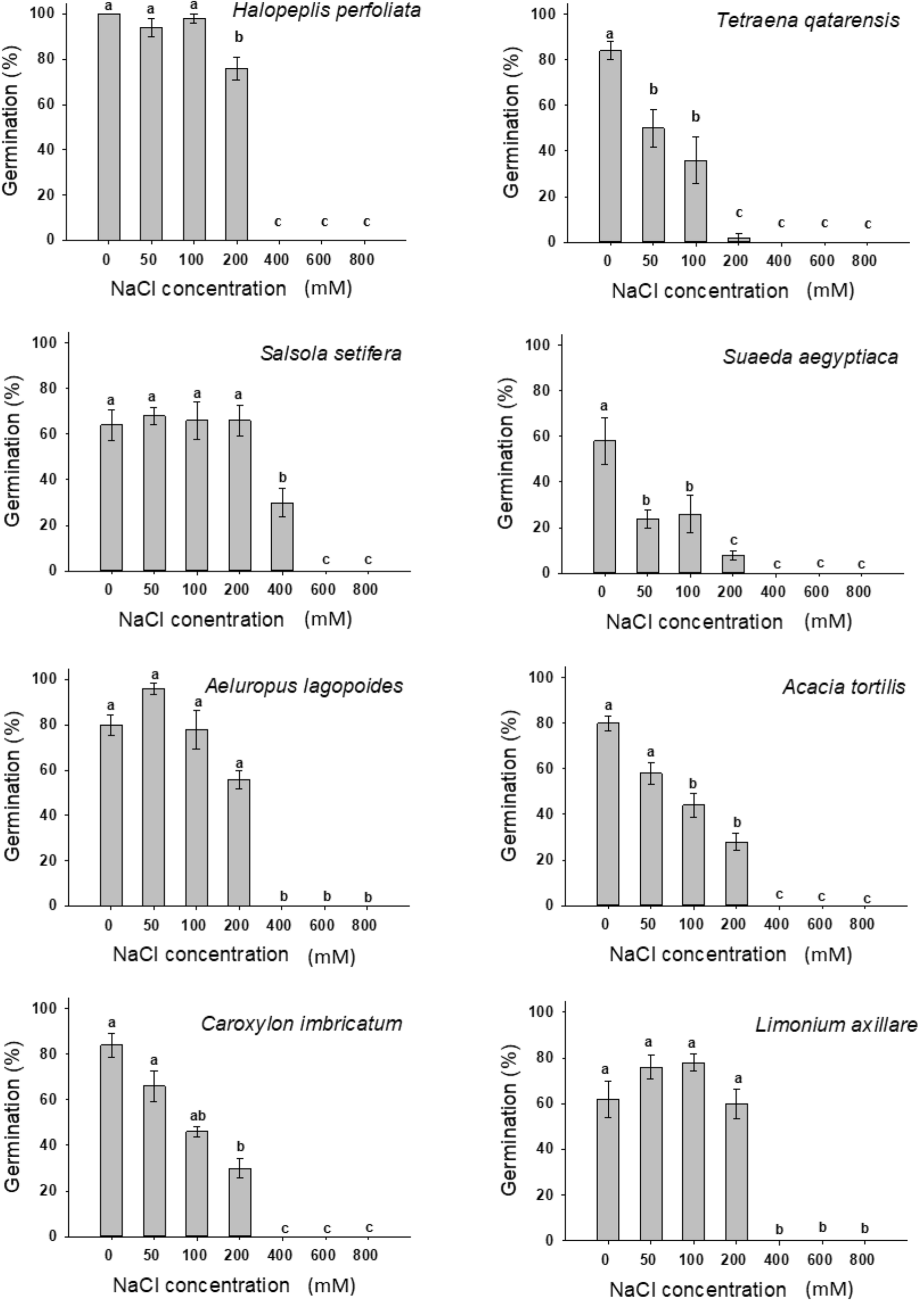
Cumulative percentage of seed germination of halophytes as dependent on NaCl concentration. Values shown are means ± SE. Values in a figure with a common letter are not significantly different according to Tukey's test at *p* ≤ 0.05.

For providing base knowledge about the seeds of halophytes collected from Qatar, the seeds were photographed and characterized (Fig. 1, Table 2). Significant effects (*p* ≤ 0.01) of the salinity factor on the germination potential of the eight studied species were found (Fig. 2). The studied species achieved a range of 30–80% germination at concentration less than 200 mM. Almost all the species did not show any germination at higher NaCl concentration (600 and 800 mM). As the salt concentration increased, a decreasing trend of final germination occurred in all species. Around 30% of seeds of *Salsola setifera* germinated at 400 mM NaCl concentration. The rate of germination was significantly affected by salinity (*p* ≤ 0.01; Table 3).

**Table 2:** Characterization of seeds for the studied species.

Species	Wt. of 100 seeds (g)	Length (mm)	Width (mm)	Color
*Acacia tortilis*	2.076	3.7 ± 0.367*	1.16 ± 0.256*	Brown/dark brown
*Caroxylon imbricata*	0.117	0.42 ± 0.091	0.39 ± 0.035	Beige color
*Tetraena qatarense*	0.033	0.83 ± 0.082	0.4 ± 0.061	Clay color
*Limonium axillare*	0.032	0.77 ± 0.065	0.3 ± 0.082	Maroon
*Sueda aegyptiaca*	0.18	0.53 ± 0.091	0.44 ± 0.075	Burgundy
*Aeluropus lagopoides*	n.a	0.5 ± 0.092	0.32 ± 0.049	Honey to apricot color
*Halopeplis perfoliata*	n.a	0.43 ± 0.062	0.33 ± 0.075	Golden brown
*Salsola setifera*	n.a	0.82 ± 0.153	0.66 ± 0.103	Dark brown to black

*Values are means ± Standard deviation (n = 10).

n.a.: data not available.

**Table 3:** Germination indices of eight halophyte species at different NaCl concentrations.

Species	0 mM	50 mM	100 mM	200 mM	400 mM	600 mM	800 mM
*H. perfoliata*	40 ± 0.0^a^*	37.6 ± 1.6^a^	39.20 ± 0.80^a^	30.40 ± 2.04^b^	0.00 ± 0.00^b^	0.0 ± 0.0^b^	0.0 ± 0.0^b^
*T. qatarensis*	35.8 ± 1.3^a^	22.4 ± 2.6^a^	15.90 ± 2.089^a^	3.5 ± 0.50^b^	0.00 ± 0.00^b^	0.0 ± 0.0^b^	0.0 ± 0.0^b^
*S. setifera*	27 ± 1.78^a^	27.5 ± 1^a^	22.5 ± 1.031^a^	17 ± 1.3^a^	8.90 ± .6^b^	0.0 ± 0.0^b^	0.0 ± 0.0^b^
*S. aegyptiaca*	7.2 ± 1.2^a^	3.3 ± 0.875^a^	2.70 ± 1.102^a^	0.90 ± 0.367^a^	0.00 ± 0.00^b^	0.0 ± 0.0^b^	0.0 ± 0.0^b^
*A. lagopoides*	14.4 ± 0.75^a^	18.8 ± 0.8^a^	15.6 ± 1.72^a^	10.8 ± 0.49^a^	0.00 ± 0.00^b^	0.0 ± 0.0^b^	0.0 ± 0.0^b^
*A. tortilis*	28 ± 1.11^a^	20.3 ± 1.72^a^	15.4 ± 1.785^a^	9.8 ± 1.31^a^	0.00 ± 0.00^b^	0.0 ± 0.0^b^	0.0 ± 0.0^b^
*C. imbricatum*	29.4 ± 1.79^a^	23.1 ± 2.37^a^	16.1 ± 0.857^a^	10.5 ± 1.565^a^	0.00 ± 0.00^b^	0.0 ± 0.0^b^	0.0 ± 0.0^b^
*L. axillare*	12 ± 1.79^a^	14.4 ± 1.33^a^	14.8 ± 1.02^a^	11.2 ± 1.02^a^	0.00 ± 0.00^b^	0.0 ± 0.0^b^	0.0 ± 0.0^b^

*Values are means ± SE (n = 5). Means in a row sharing the same 1etter are not significantly different according to Tukey's test at *p* ≤ 0.05.

**Figure 3:**
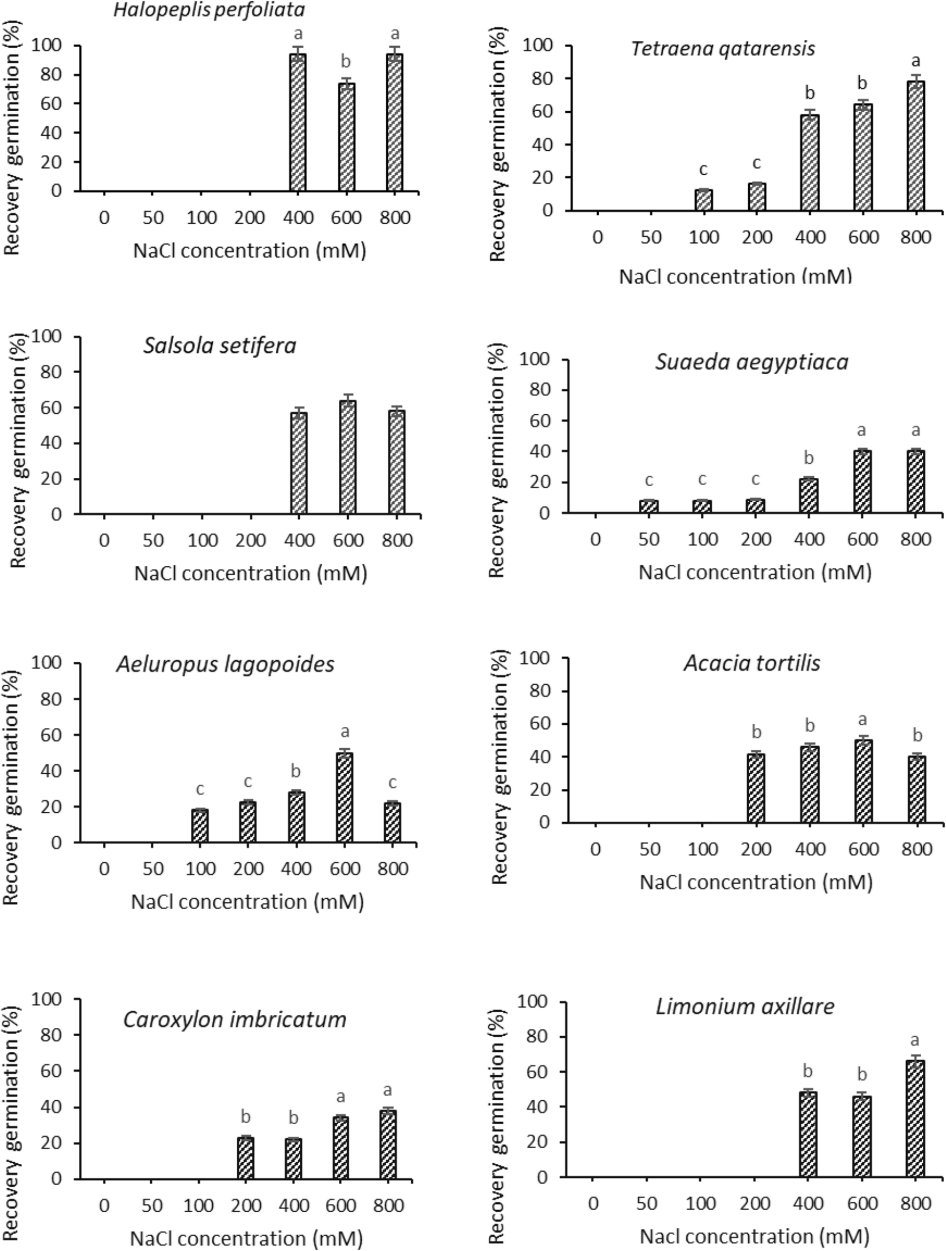
Percentage of seeds that able to recover germination when subjected to distilled water after three weeks of NaCl treatments. Values shown are means ± S.E. Values in a figure with a common letter are not significantly different according to Tukey's test at *p* ≤ 0.05. The absence of letters in a graph means no significance among treatments.

For *Halopeplis perfoliata,* Timson’s index values ranged from low values of zero (0) for the 400, 600 and 800 mM salt treatments to a mean high value of 40 (out of 50, the highest possible value) for the control treatment (0 mM). *H. perfoliata* showed high germination percentage (100, 94, 98% and 76% at 0, 50, 100 and 200 mM NaCl respectively), however germination dropped significantly to 0% at the higher concentrations. Figure 3 shows that non-germinated seeds from 400 and 600 mM treatments recovered when salt stress conditions were alleviated.

For *Tetraena qatarense*, the seed germination was significantly reduced under salt treatments compared with 0 mM NaCl control treatment, however no significant differences occurred between 50 and 100 mM and less than 3% of seeds germinated at 200 mM NaCl treatment (Fig. 2). The recovery percentage increased as the pretreatment concentration of salinity increased from 12% at 100 mM to 78% at 800 mM (Fig. 3). For *Salsola setifera* seeds, the germination was around 65% at control and up to 200 mM NaCl concentration with no significant difference among treatments (Fig. 2). At 400 mM the germination percentage was 30% and then dropped to 0% at higher NaCl concentrations. The recovery germination percentage was ca. 60% at 400, 600 and 800 mM, respectively (Fig. 3).

The seed germination of *Suaeda aegyptiaca* seeds decreased from 58% at 0 mM to 8% at 200 mM and no seeds germinated at 400 mM and above concentrations (Fig. 2).

The maximum seed germination achieved for *Aeluropus lagopoides* seeds was 96% obtained at 50 mM concentration and then declined to 78% at 100 mM and to 56% at 200 mM NaCl (Fig. 2). The recovery was the highest at 600 mM and achieved 50% (Fig. 3). For *Acacia tortilis* seeds, the highest germination was 80% at control treatment and 28% of seeds germinated at 200 mM NaCl then the germination dropped significantly to 0% at higher concentrations (Fig. 2) and 50% of seeds recovered after pretreatment with 600 mM NaCl (Fig. 3). Similarly, no seeds of *Caroxylon imbricatum* were germinated at concentration above 200 mM NaCl (Fig. 2) and the recovery of germination ranged between 22% at (400 and 200 mM), 34% at (600 mM) and 38% at (800 mM NaCl) (Fig. 2). For *Limonium axillare* seeds, the germination reached 78% at 100 mM and no seed germinated at higher concentration than 200 mM NaCl (Fig. 2). The recovery percentage was 66% at 800 mM NaCl (Fig. 3).

### Seedling experiment

**Figure 4:**
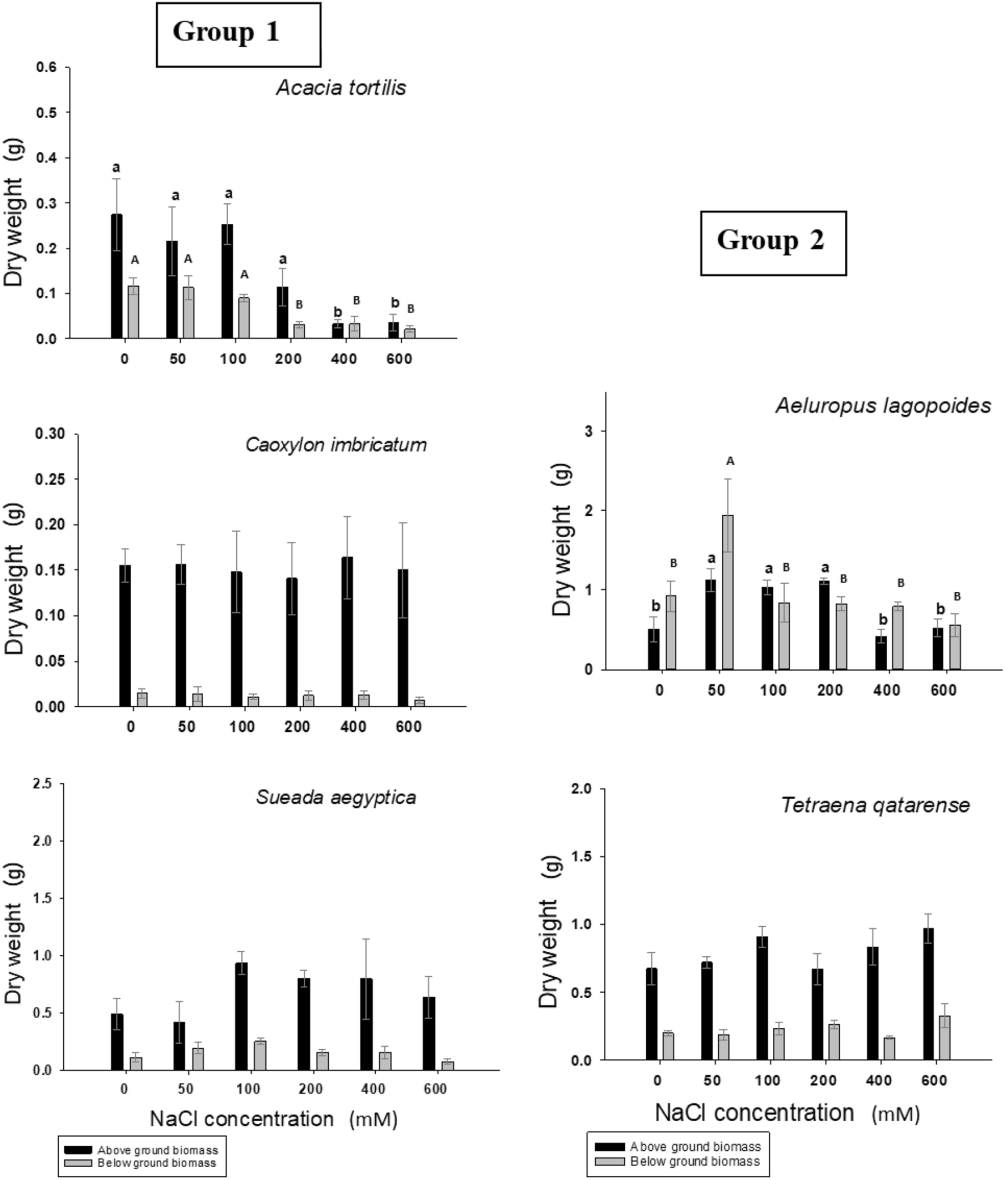
Dry weight of seedling under the influence of different NaCl concentrations. Values shown are means ± S.E). Values in a figure with a common letter (small for aboveground, capital for belowground biomass) are not significantly different according to Tukey's test at (*p* ≤ 0.05). Group-1 refers to plants that had more than 50% germination at concentration equal or less than 200 mM NaCl while Group-2 represents plants that had more than 50% germination at concentration of equal or less than 50 mM NaCl. The absence of letters in a graph means no significance among treatments.

**Figure 5:**
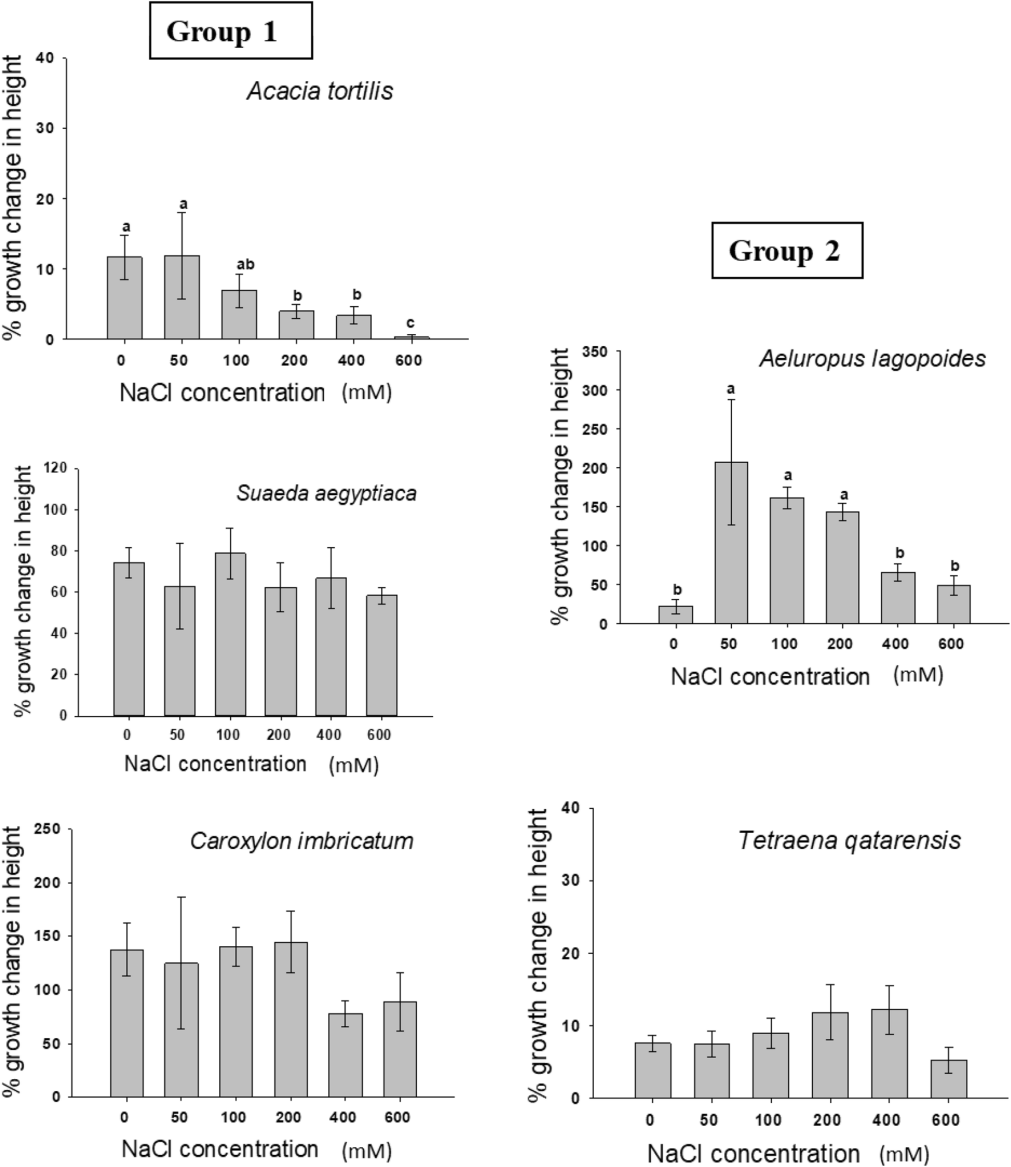
Percent of growth change in height of seedling under the influence of different NaCl concentrations. Values shown are means ± SE. Values in a figure with a common letter are not significantly different according to Tukey's test at (*p* ≤ 0.05). Group-1 refers to plants that had more than 50% germination at concentration equal or less than 200 mM NaCl while Group-2 represents plants that had more than 50% germination at concentration of equal or less than 50 mM NaCl. The absence of letters in a graph means no significance among treatments.

Six-to-eight-week-old seedlings of *Tetraena qatarense, Sueada aegyptica and Caroxylon imbricatum* showed no significant change in biomass (dry matter, g/plant) of both above and belowground parts after being subjected to different saline treatments (0–600 mM NaCl) (Fig. 4). The dry weight of *A. lagopoides* was significantly increased (*p* ≤ 0.05) at 50 mM NaCl compared to control (0 mM NaCl) treatment, however the plants showed similar growth to control treatment when treated with salt concentration up to 600 mM NaCl. *Acacia tortilis,* although it can grow similar to control treatment up to 200 mM NaCl, showed a significant low biomass at 400 and 600 mM. The response of the aboveground and belowground biomass to salinity gradient had similar trends in all studied species (Figs. 5) except *A. lagopoides* where the aboveground exerted a maximum significant increase in growth at 50 mM NaCl while the belowground shown an even increase at concentrations from 50 to 200 mM NaCl (Fig. 4).

The post-treatment plant height was recorded for each plant after 6 weeks of salinity treatments. The percent change for the height of *Tetraena qatarense*, *Sueada aegyptica and Caroxylon imbricatum* showed no significant differences among all treatments at *p* ≤ 0.05 (Fig. 5). In *Acacia tortilis* there was a significant decrease in in height with increasing salinity (*p* ≤ 0.05; Fig. 5). For *Aeluropus lagopoides* the percent change in height at 50, 100 and 200 mM of NaCl was higher than control, 400 and 600 mM treatments (Fig. 5) without significant differences in growth change in height among control, 400 and 600 mM NaCl concentrations (Fig. 5).

## Discussion

In the present study, the results showed that seeds of different species responded differently to salinity gradient and the increased concentration of salt treatment generally decreased the germination percentage and germination rate of all studied species. Previous studies have reported similar findings for other halophyte species^32–35^. The studied species can be divided into two groups: (1) halophytes that had more than 50% germination at concentration equal or less than 200 mM NaCl, which include: *Halopeplis perfoliata*, *Salsola setifera*, *Aeluropus lagopodes* and *Limonium axillare,* and (2) halophyte species that had more than 50% germination at concentration of equal or less than 50 mM NaCl, which include *Tetraena qatarense*, *Caroxylon imbricatum* and *Acacia tortilis*. Many studies on seed germination of halophytes reported that the halophyte seeds germinated well in non-saline water or at salinity less than 100 mM of sodium chloride^21,24,35^. Furthermore, the degree of salt tolerance during the germination stage of the seeds varies among halophyte species. Some halophyte seeds have the ability to germinate in a concentration up to 1300 mM (e.g. *Haloxylon ammodendron*^36^) and others up to 1700 mM (e.g. *Salicornia herbacea*^37^) On the other hand, certain halophyte seeds were reported to fail to germinate on a concentration more than 250 mM NaCl, among those *Salicornia brachystachya*^38^. Moreover, many halophytes can tolerate salt on their mature plant form, but their seeds still failed to germinate on concentration of above 300 mM NaCl^39^.

Similar to what obtained by^32,40,41^, seeds of *Halopeplis perfoliata* were not dormant and ready to germinate at the control treatment (Fig. 2). In the present study, *H. perfoliata* seeds achieved similar germination in distilled water, but failed to germinate at higher treatments (e.g. 400 mM). Similar to our results, in Saudi Arabia, the ability of seeds of *H. perfoliata* were failed to germinate at concentration more than 250 mM^32^. While El-Keblawy & Bhatt (2015) reported germination at 400 mM NaCl^40^. Exposure to high salinity not only reduced the total germination percentage, but also reduced the germination rate (Table 3). The responses of recovery of seeds vary among halophyte^25^. Some species like seeds of the salt marsh halophyte *H. perfoliata* showed high (80%) recovery when transferred from hyper-salinity (2.0 M NaCl) to distilled water and also like *Salsola imberica* which has been reported to recover from high salinity treatment up to 1 M NaCl^25^, while other, like *Cressa cretica*, showed less germination percentage of recovery at concentration more than 0.4 M NaCl^42–44^. This enforced dormancy due to salinity is a key adaptive feature of halophyte seeds that maintain a persistent soil seed bank so that seedling recruitment is expected after sufficient rain^31,37,45^.

*H. perfoliata* is a succulent halophyte, which is known to inhabit coastal salt marshes of the Arabian Peninsula and considered as an important intertidal terrestrial producer upon which food webs can be established^46^. The plant has potential economic uses in soap and glass manufacturing industries^47^ and can serve in ecological restoration as sand dune stabilization in coastal deserts.

Germination of seeds of *T. qatarense* was found to decrease with salinity and temperature increase^48^. Germination percentage of *Z. album* in the Western coastal habitat in Egypt was highest (91.7%) at 0 mM and lowest (7.7%) at 400 mM NaCl^49^. Results about another relative species, the succulent desert annual, *Z. simplex*, in Pakistan, indicated that germination was failed to occur at concentration of more than 100 mM NaCl^26^. In the present study*,* exposure to high salinity is not only reduced the germination percentage, of *T. qatarense* but also reduced the overall germination rate (Table 3). Similar results were recorded for *Z. simplex*^26^. Other studies reported that *Z. simplex* recovery was only 20% after a salt treatment of 125 mM NaCl^26^. *T. qatarensis* inhabits inland salt flats and coastal sandy and rocky shores in Qatar^17^.

Soleimani et al. (2011) indicated that *Anabasis aphylla* can tolerate salt treatment and it is good to be irrigated with salt water up to 300 mM NaCl^50^. *Salsola setifera* is classified as a dwarf succulent shrub inhabits inland salt flats and coastal sandy and rocky shores in Qatar^17^. As a succulent plant, high internal salt levels are compensated by high water storage and excess salt is converted to crystals in the chlorenchyma tissue with increase in plant succulence as both water and salt absorption increases^17^. The plant can be a good fodder for livestock especially during summer^51^ and has been reported to produce volatile compounds that contain 85% of carvacrol and 15% thymol^52^.

According to Ungar (1991), around 26% of seeds of the leaf succulent halophytes (like *Suaeda aegyptiaca*) failed to germinate at concentrations above 200 mM NaCl and no recovery was obtained from another *Suaeda* species, *S. fruticose*^35,53^. *S. aegyptiaca* is classified as asucculent shrub inhabits coastal sandy and rocky shores in Qatar^17^. It could be used as either forage or fodder, fuel wood, as well as a source of large quantities of carbonate of soda that can be used for the soap and glass industry^51^.

For the germination of *Aeluropus lagopodes*, our findings indicate higher germination than what was obtained by Joshi et al. (2005) in India, who found that no seed germination occurred at concentration of 86 mM or higher^34^. In Pakistan, Gulzar and Khan (2001) reported that the seeds of *A. lagopoides* have no dormancy and the germination can reach 100% in the non-saline water^33^. In the present study, it lasted around 12.2 days to reach 50% germination in distilled water. This may be due to other factors that interact in seed germination, like temperature and species genetic diversity^34^. In Qatar, *Aeluropus lagopoides* inhabits inland wetlands, inland salt flats, coastal high marshes, and coastal sandy shore habitats^17^. The plant is characterized by thick cuticles and a cover of waxy layers with hairs on leaves and stems, their seeds remain dormant at a high salinity, and recover when returned to distilled water^51^. The plant possess forage economical values and known to be as traditional fodder of arid regions^51^. Germination percentage for *Acacia tortilis* seeds was the highest reaching 80% of germination and seeds of *A. tortilis* can germinate at concentration up to 200 mM NaCl. Recovery was the highest for seeds that have been pre-treated (Fig. 3). Abari et al. (2011) found that *A. tortilis* and *A. nilotica* are salt tolerant plants and might germinated up to 300 mM NaCl.

Germination percentage for *Caroxylon imbricatum* seeds (previously known as *Salsola imbricata*) was the highest at distilled water(Fig. 3). Our results are around 10% more than what have been recorded in UAE^54^. They found that seeds of *C. imbricatum* germinated at 500 mM NaCl and the germination was completely inhibited at 600 mm NaCl. In Kuwait, Zaman et al. (2010) recorded that the germination was significantly declined on 400 and 600 mM NaCl with a value of less than 10% and completely inhibited at 800 mM NaCl^55^. In Pakistan, *Salsola imbricata* germinated on a concentration of up to 800 mM^56^. In the present study, the germination was completely inhibited lesser concentration. The highest germination recovery was around 40% (Fig. 3). However, there was no recovery recorded by Mehrunnisa et al., (2007) at 800 mM. It can be realized that there were variability on responses of the *C. imbricata* seeds to salinity gradient which might be due to genetic diversity or effect of local environment. *C. imbricatum* is classified as dwarf succulent shrub inhabits inland salt flats and coastal sandy and rocky shores in Qatar^17^. The plants are traditionally known to provide relief in diseases like cold, flu and cough and skin diseases^51^.

Interestingly, the highest germination percentage for *Limonium axillare* was at salinity treatment instead of distilled water (Fig. 2). Data presented by Mahmoud et al. (1983) indicated that the seed germination reached only 10% at 170 mM NaCl, however in the present study, a higher germination was reached. It took around 2.6 days to reach 50% germination at 100 mM concentration. This rapid germination was also recorded for another species of *Limonium*, *L. stocksii*^27^. *L. axillare* inhabits high marshes and coastal sandy-rocky shores of Qatar^17^. The plant is a well-adapted halophyte and known to have well developed salt glands^17,18^. High concentrations of organic compounds (i.e. proline, soluble sugars, nitrogen, and photosynthetic pigments) and salts (Na^+^, Cl^−^, and Ca^2+^) were found in their tissues^18^.

For seedling growth, the effect of salinity on overall growth varies according to literatures^1,57^. Halophytes respond differently toward increasing salinity from stimulation to inhibition of growth^1^. In general, many dicotyledonous halophytes showed optimal growth at concentrations ranged from 50 to 250 mM NaCl^58^ while monocotyledonous halophytes showed optimal growth at concentration less than 50 mM NaCl^59^. In the present study, seedlings of *Tetraena qatarense, Suaeda aegyptica and Caroxylon imbricatum* showed no reduction in biomass, including both aboveground and belowground dry weight, against the salinity gradients up to 600 mM NaCl. Analyzing of the percent change in plant height during the period of experiment for *T. qatarense, S. aegyptica and C. imbricatum* indicated no significant differences on height among salinity treatments and control. Available records about seedling growth of other species belong to genus *Suaeda, S. maritima*^60^ and S. *physophora*^61^ indicated that the growth become well in the presence of salt. These results indicate that the *Suaeda* species are potential candidates for growing halophyte crops for different applications i.e. forages, greenery, biomass, bio-products and others.

Our study pointed out that the seedlings of *A. lagopoides* tended to grow better with treatment than regular tap water. Moreover, the growth remained similar to that of control treatment at 600 mM NaCl treatment. The change in height for *A. lagopoides* was higher at lower concentrations compared with control and concentrations higher than 200 mM NaCl treatments. This may explain the success of abundance of this species in Sabkhas (saline soil) habitats of Qatar. Coincide with our results, Joshi et al., (2005) pointed out seedlings of *A. lagopoides* need to be raised first in low concentrations of seawater before transplanting them onto wastelands having high saline concentrations^34^.

Although *Acacia tortilis* is not classified as halophyte due to the non-highly saline natural habitats, it was described as highly salt-tolerant plant. In the present study, *A. tortilis* seedling can grow up with NaCl concentration, but it showed a significant less biomass with prominent leaf shedding. Leaf shedding is one of the mechanisms of halophytes to get rid of accumulative effect of salt^29,62^. The effect of salinity on seedling of *A. tortilis and A. nilotica* at three concentrations (0, 150 and 300 mM) was studied by Mehari et al., (2005) who found that both species responding in similar manner to salinity^63^. They suggested that the two species are suitable for growth on saline affected soils.

In conclusions, halophytes recently play important roles for restoration of coastal ecosystems and for future saline agriculture. The results of this research provide baseline information for future research in exploring economic benefits of halophytes in the State of Qatar. Halophytes had different responses to salt stress at seed germination stage. Differences in responses to salt concentration were also observed between Qatar and other countries’ intraspecific halophyte populations. Except for *Salsola setifera* all the seven species failed to germinate on concentration of more than 200 mM NaCl. When the seeds were exposed to higher concentration of salts, they became more ready to germinate in freshwater treatment. This may indicate that salt stress exposure might be a stimulator of germination if followed by freshwater exposure. Experiments on seedling growth of *Tetraena qatarense, Suaeda aegyptica and Caroxylon imbricatum* showed no growth reduction after saline water irrigation up to 600 mM NaCl, therefore the three species are potential candidates for growing halophytic crops for different economical and/or environmental benefits.

Thus, the halophytes examined in this study would be of considerable economic value for improvement of the affected saline soils and increasing yield productivity in coastal areas. However, in order to evaluate the feasibility of halophytes to be stand as a commercial agricultural crop, forage crop or for other ecological or commercial purposes, further studies should be conducted under field conditions and for the entire growing season with different saline water irrigation regimes. The long-term well-monitored field studies are necessary prior to any made decision about long-scale halophyte agriculture.
